# Continuous white-light/indocyanine green overlay endoscopy in pituitary adenoma surgery: A prospective feasibility and safety study

**DOI:** 10.1016/j.bas.2026.106144

**Published:** 2026-06-23

**Authors:** Ida Olesrud, Martin K. Andersen, Christopher Larsson, Ansgar Heck, Daniel Dahlberg, Markus K.H. Wiedmann

**Affiliations:** a Institute of Clinical Medicine, Faculty of Medicine, University of Oslo, Norway; b Department of Neurosurgery, Oslo University Hospital, Norway; c Department of Radiology, Diakonhjemmet Hospital, Oslo, Norway; d Department of Endocrinology, Oslo University Hospital, Norway

**Keywords:** Pituitary adenoma, Endoscopic surgery, Fluorescence guided surgery, Indocyanine green, Intraoperative fluorescence

## Abstract

**Introduction:**

Intraoperative differentiation between pituitary adenoma (PA) and normal gland tissue remains a key challenge in endoscopic transsphenoidal surgery (ETSS). Indocyanine green fluorescence (ICG-FL) has been proposed as a visual adjunct. Existing data are limited and primarily based on first-generation systems requiring intermittent mode switching.

**Research question:**

Is implementation of a second-generation ICG endoscope with continuous white-light/near-infrared (WL/NIR) overlay safe and feasible in routine PA surgery?

**Materials and methods:**

This prospective single-center observational study included consecutive patients undergoing ETSS for PA over a 28-month period. A second-generation ICG-FL endoscope with continuous WL/NIR overlay visualization was used throughout tumor resection. Primary endpoints were adverse events related to ICG-administration or device use. Secondary endpoints included surgical complications, short-term endocrine outcome and radiological residual tumor.

**Results:**

After screening, 130 patients met inclusion criteria. Continuous WL/NIR overlay was successfully applied in all cases without intraoperative technical failure, dye-related adverse events, or need to revert to white-light-only visualization. Among functional PAs, 75% (52/69) achieved early biochemical remission. At follow-up, 22 patients (17%) had radiologically confirmed residual tumor, while 73 (56%) showed no residual disease. When combined with functional cases not undergoing MRI due to confirmed remission (n = 27), extrapolated gross-total resection was 77%.

**Discussion and conclusions:**

Continuous ICG-FL guidance was safe and feasible in routine ETSS for PA, without compromising surgical workflow, anatomical visualization, or requiring conversion to white-light-only endoscopy. Complication rates were consistent with contemporary endoscopic series. Continuous fluorescence provides real-time perfusion information that may facilitate improved tissue differentiation and warrants further comparative evaluation.

## Highlights

• Continuous WL/NIR ICG overlay was feasible in 130 pituitary adenoma cases.
• No ICG - or device-related adverse events or technical failures occurred.
• Overlay endoscopy did not require conversion to white-light-only mode.
• Surgical morbidity and early endocrine outcomes matched contemporary series.
• These data support future comparative studies of fluorescence guidance.

## 1 Introduction

Although generally histologically benign, pituitary adenomas (PAs) can displace or infiltrate adjacent structures, including the normal pituitary gland, cavernous sinus, and diaphragma sellae (Asa et al., 2022). Clinical presentation depends on hormonal activity and local mass effect (Asa et al., 2022; Tritos and Miller, 2023). For most symptomatic lesions, surgery remains the primary curative treatment (Tritos and Miller, 2023). However, reported rates of gross-total resection (GTR) and endocrine remission vary widely across series (Almeida et al., 2018; Bengtsson et al., 2023; Falch et al., 2023; Nieman et al., 2008; Pedersen et al., 2022). Remission rates are further influenced by the criteria applied to determine biochemical remission (Nieman et al., 2008).

Surgical outcomes can be influenced by several factors, including tumor size- and volume, cavernous sinus invasion (Knosp-grading) and repeat surgery (Almeida et al., 2018; Castle-Kirszbaum et al., 2023; Knosp et al., 1993; Lobatto et al., 2018; Micko et al., 2015). Achieving the optimal balance between surgical radicality and preservation of pituitary function represents a complex issue in pituitary surgery. Obtaining GTR can be particularly demanding in cavernous sinus–invasive tumors (Knosp grade 3–4) and recurrent disease (Almeida et al., 2018; Castle-Kirszbaum et al., 2023). Inadequate resection may result in persistent hypersecretion or tumor recurrence, whereas excessive resection increases the risk of postoperative hypopituitarism. Intraoperative tumor identification currently relies predominantly on visual cues under white-light endoscopy, supplemented by tactile feedback and tissue consistency. These methods may be insufficient in cases with distorted anatomy or scarring. This limitation has driven interest in intraoperative fluorescence techniques aimed at enhancing tissue contrast and improving real-time differentiation between PA and normal gland (Catapano et al., 2018; Litvack et al., 2012; Olesrud et al., 2025).

Indocyanine green (ICG) fluorescence is increasingly used in neurosurgery for visualization of tumor and vascular structures. ICG binds to albumin and emits fluorescence under near-infrared light (NIR). Based on differential tissue perfusion, the normally vascularized pituitary gland is expected to demonstrate stronger ICG fluorescence than adenoma tissue, a concept first proposed for endoscopic transsphenoidal surgery (ETSS) more than a decade ago (Litvack et al., 2012). Subsequent case reports have demonstrated feasibility, but available evidence is limited to small, heterogeneous series, predominantly using first-generation endoscopes that require switching between white-light (WL) and NIR optical modalities, precluding true continuous fluorescence-guided resection (Catapano et al., 2018; Olesrud et al., 2025; Muto et al., 2023; Verstegen et al., 2016).

Second-generation ICG-endoscopes integrate standard WL endoscopy with NIR visualization of ICG fluorescence in an overlay mode. This NIR/WL overlay enables continuous visualization of ICG fluorescence superimposed on the standard WL image, allowing simultaneous and uninterrupted visualization of both modalities throughout tumor resection. Despite this technical advance, clinical data addressing the implementation, safety, and feasibility of continuous NIR/WL overlay ICG visualization in pituitary surgery remain limited.

Before outcome-driven comparative studies can be meaningfully pursued, the practical integration and safety of this strategy must be established. Accordingly, this study evaluates the implementation, safety, and feasibility of continuous ICG-fluorescence visualization during ETSS for PAs at a tertiary referral center.

## 2 Methods

### 2.1 Study design

This is a prospective single-center observational study including consecutive patients undergoing ETSS for PA at Oslo University Hospital (OUH). The primary endpoint was the occurrence of adverse events related to ICG-administration, or use of the NIR/WL overlay endoscope. Secondary endpoints included surgical complications, presence of radiological residual tumor, remission for functional PAs, and new pituitary insufficiency at radiological and endocrinological follow-up at approximately three months postoperatively. Secondary endpoints were included to contextualize feasibility within routine clinical outcomes, not to assess comparative efficacy. Feasibility was defined as successful integration of continuous WL/NIR overlay visualization into routine surgical workflow, including uninterrupted use throughout tumor resection, absence of technical failure, absence of workflow-disrupting events, and no requirement for conversion to white-light-only visualization.

### 2.2 Patient population

**Fig. 1:**
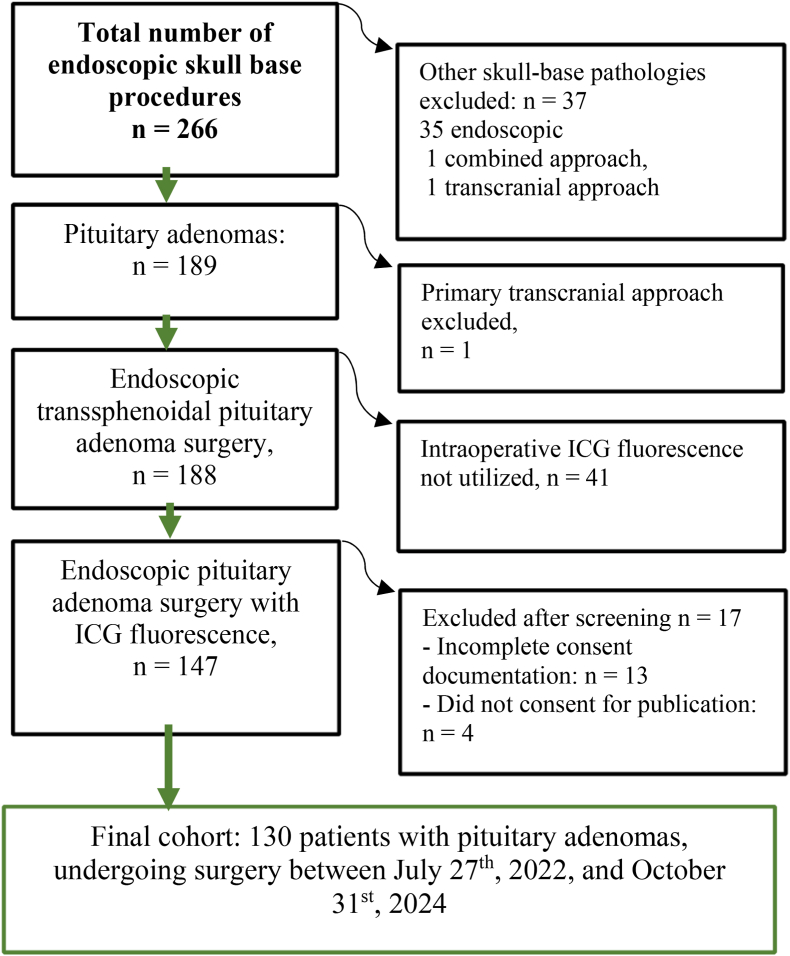
Flow diagram illustrating patient selection and derivation of the final ICG-study cohort.

All patients undergoing ETSS for pituitary tumors between July 1, 2022, and October 31, 2024, were screened for inclusion. Patients were eligible if they had a histologically verified PA, underwent surgery with documented ICG administration, and provided written informed consent for study participation and data publication. During the implementation period, not all pituitary adenoma procedures were performed by surgeons participating in the fluorescence program. Consequently, a subset of patients underwent standard endoscopic surgery without ICG administration. In our center, case allocation is largely determined by administrators, based on surgeon availability and service logistics, rather than patient- or tumor characteristics. Procedures for other skull-base pathologies, combined transcranial approaches, and cases without ICG use were excluded (Fig. 1).

### 2.3 Data collection

Perioperative and follow-up variables were prospectively entered into the institutional pituitary registry. Registry entries were cross-checked against operative schedules and electronic medical records (procedure codes AAE00/AAE10, NOMESCO Classification of Surgical Procedures). Missing or incomplete data were supplemented by structured chart review performed by an independent observer not directly involved in the surgeries. Only patients with a complete set of signed consent forms were included in the final cohort.

### 2.4 Imaging and radiological assessment

Preoperative MRI measurements (two-dimensional) were performed in coronal and sagittal planes according to Øystese et al. (2019). Volumetric tumor assessment was performed using summation of slices (Brainlab Elements®). Cavernous sinus invasion was graded using the Knosp classification (Knosp et al., 1993). Immediate postoperative MRI and follow-up MRI at approximately three months were evaluated for residual tumor based on radiological reports. In cases of ambiguous radiological reports, images were re-reviewed by an independent radiologist blinded to endocrine outcome, with senior neuroradiology consensus when required.

### 2.5 Surgical technique and ICG administration

**Fig. 2:**
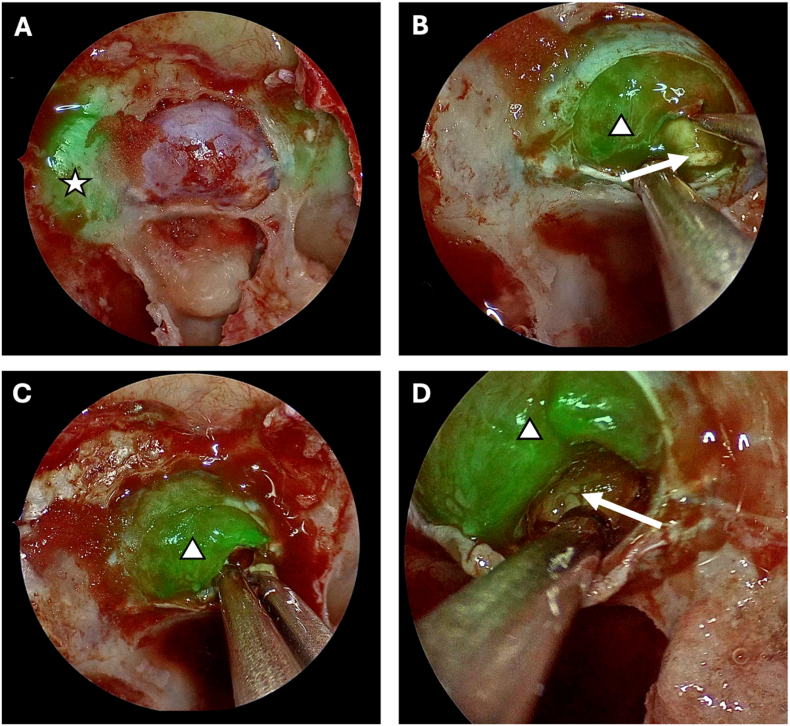
a–d. Representative intraoperative images demonstrating continuous WL/NIR ICG overlay fluorescence during pituitary adenoma resection for acromegaly. (a) Initial appearance of ICG fluorescence within the internal carotid artery (asterisk), defining baseline time (0 min) after intravenous administration. (b) Six minutes after baseline, exposed intrasellar contents demonstrating differential fluorescence between presumed pituitary gland tissue (white triangle) and underlying adenoma (white arrow). (c) Sixteen minutes after baseline, ongoing tumor resection under continuous overlay fluorescence with persistent signal in adjacent pituitary gland tissue. (d) Twenty-6 min after baseline, removal of residual non-fluorescent tissue. Time-stamped biopsy specimens were collected throughout the procedure and were histopathologically verified as adenoma or normal pituitary tissue.

All procedures were performed using an uni- or binostril four-hand endoscopic transsphenoidal technique. A second-generation NIR/WL overlay endoscopic system was used in all cases, consisting of the IMAGE1 S™ Rubina camera platform and a 4-mm, 0° telescope (KARL STORZ). ICG was administered intravenously after sellar dura exposure. A standard bolus dose of 10 mg was adopted during the study period; additional boluses were given selectively at the surgeon's discretion. Representative intraoperative images demonstrating continuous NIR/WL overlay ICG fluorescence during tumor resection are shown in Fig. 2. Fluorescence signals were interpreted qualitatively by the operating surgeons in the context of anatomical landmarks and surgical progression; no predefined fluorescence intensity thresholds were applied.

### 2.6 Endocrine outcome

Approximately three months after pituitary surgery, the patients underwent endocrine follow-up including evaluation for hormone deficiencies and excess hormone secretion. For acromegaly, criteria for remission was growth hormone (GH) suppression (GH nadir <0.4 μg/l) during oral glucose tolerance test (OGTT) or normalized age adjusted insulin-like growth factor (IGF-1) at the 3-month mark, according to recent guidelines (Melmed et al., 2025). In prolactinomas, remission was based on 3-month prolactin level within/below adjusted normal range (Petersenn et al., 2023). In Cushing's disease, remission was defined as a nadir plasma cortisol value < 138 nmol/L measured within the first 7 postoperative days (Nieman et al., 2008). New postoperative pituitary insufficiency was defined as a novel deficit requiring hormonal substitution at endocrinological 3-month follow-up. Cortisol replacement in Cushing cases was not considered a novel failure. Three-month assessment was selected, as this is the standardized early postoperative evaluation point used in our institutional pituitary follow-up pathway.

### 2.7 Complications

Complications were recorded prospectively in the institutional pituitary registry and subsequently verified by independent chart review. Relevant surgical complications were included, also when occurring beyond 30 days. Recorded events comprised cerebrospinal fluid (CSF) leak requiring intervention, meningitis (defined as antibiotic treatment for clinically suspected and/or biochemically verified central nervous system infection), epistaxis, postoperative hemorrhage, vascular events, transient arginine vasopressin deficiency (AVP-D), and syndrome of inappropriate antidiuretic hormone secretion (SIADH). Nasal morbidity, including sinusitis and sinonasal symptoms, was not systematically assessed.

### 2.8 Statistical analysis

Analyses were performed using SPSS v30 and RStudio. Descriptive statistics are reported. No formal power analysis was performed as the study was not designed to test a predefined efficacy hypothesis or compare outcomes between treatment groups. Rather, the study had an observational feasibility and safety design aimed at evaluating implementation of a surgical visualization technology in routine clinical practice.

### 2.9 Ethical and Regulatory approvals

The study was conducted in accordance with the Declaration of Helsinki (Association, 2025). Written informed consent for intraoperative ICG administration and inclusion of clinical data for research and publication was obtained from all patients. The study protocol was approved by the Regional Committee for Medical and Health Research Ethics (REK no. 399198) and the institutional Privacy Protection Officer at Oslo University Hospital (PVO no. 22/21929).

Because the NIR/WL overlay endoscope was not certified under the European Medical Device Regulation (EU, 2017/745), an exemption from the conformity assessment procedure was granted by the Norwegian Medicines Agency (NoMA, ref. 22/09780-2) In accordance with this approval, separate written consent for the use of non-CE-marked equipment was obtained for all patients undergoing endoscopic transsphenoidal surgery during the study period, independent of study participation.

## 3 Results

### 3.1 Study population

During the study period, 188 consecutive patients underwent ETSS for PAs. Intraoperative ICG fluorescence was used in 147 cases, leading to exclusion of 41 non-ICG cases. An additional 17 ICG cases were excluded (4 declined inclusion in publications, 13 lacked physical consent forms at time of inclusion) leaving 130 consecutive patients for final analysis (Fig. 1).

### 3.2 Intraoperative use of continuous ICG fluorescence

Continuous NIR/WL overlay visualization of ICG fluorescence was used in all included cases. No intraoperative technical failures occurred, and no anaphylactic or other adverse events related to ICG administration were observed. An initial bolus dose of 10 mg ICG was used in the majority of cases (n = 117) and provided consistent fluorescence. Additional boluses (5-10 mg) were administered selectively at the surgeon's discretion in a small number of cases (n = 11). In a minority of cases, a 5 mg bolus was administered once (n = 5) or twice (n = 4). Additional boluses were generally administered during prolonged procedures, when fluorescence intensity had diminished, or when repeat perfusion assessment of surgical or reconstructive tissues was considered useful. No adverse events attributable to repeat ICG administration were identified among patients receiving additional boluses.

Review of medical records revealed no adverse events directly attributable to the endoscope or ICG-administration. This was corroborated by the primary operating surgeons, who did not observe any such events intraoperatively. No postoperative allergic reactions to ICG occurred according to available charts. Key safety, feasibility, and clinical outcomes are summarized in Table 1.

**Table 1:** Key safety, feasibility, and clinical outcomes.

Outcome category	Result
**ICG-related adverse events**	0
Anaphylaxis or allergic reaction	0
Technical failure of overlay system	0
Need to revert to white-light-only mode	0
**Surgical complications**	
CSF leak requiring surgical repair	7 (5.4%)
Meningitis, all cases (clinical and/or biochemically verified)	7 (5.4%)
Epistaxis requiring intervention	1 (0.8%)
**Endocrine outcomes (3 months)**	
New pituitary insufficiency (any)	19 (15%)
New AVP-D	9 (7%)
Complete postoperative pituitary failure	2 (1.5%)
**Radiological outcome (≈3 months)**	
No residual tumor	73 (56%)
Definite residual tumor	22 (17%)
**Functional adenomas (≈ 3 months, n=69)**	
Biochemical remission	45 (65%)

### 3.3 Baseline patient characteristics

Baseline patient and tumor characteristics are summarized in Table 2. Mean age at surgery was 51 years (median 56, range 14 – 88 years), and 56% of patients were female. Follow-up ranged from 8 to 35 months. One patient died approximately three months postoperatively. Re-operations due to functional residual tumor activity or growth accounted for 20% (n = 26) of procedures. Functional (n = 69) and non-functional (n = 61) adenomas were quite evenly represented. Acromegaly was the most common hypersecretory syndrome (n = 42), followed by Cushing's disease (n = 17). Other functional tumors (n = 10) included prolactinomas (n = 7), TSH-producing tumors (n = 2) and a single gonadotropin producing tumor.

**Table 2:** Baseline characteristics of the study cohort.

Number of patients	n = 130	NFPA (n = 61)	FPA (n = 69)
**Preoperative patient characteristics**			
Age, Mean (range)	51 (14-88)	58 (27-88)	44.5 (14-77)
Follow-up (Months, Mean)	21	21	21
Gender			
- Male	57 (44%)	30 (49%)	42 (61%)
- Female	73 (56%)	31 (50%)	42 (39%)
Primary surgery	104 (80%)	47 (77%)	57 (82.5%)
Repeat surgery	26 (20%)	14 (23%)	12 (17.5%)
Preop. Pituitary failure/Hormone substitution (n = 129)	40 (31%)	32 (52.5%)	8 (11.5%)
**Preoperative tumor characteristics**			
Mean tumor diameter; measurement of single largest plane (range)	20 mm (0-52 mm)	28 mm (15-52 mm)	14 mm (0-45 mm)
Mean tumor volume (n = 128) (Range)	5.1 ml (0-29.6 ml)	8.6 ml (1.1-29.6)	2.1 ml (0-19.5)
Microadenomas (<10 mm)	30 (23%)	-	30 (43.5%)
Macroadenomas (≥10 mm)	100 (77%)	61 (100%)	39 (65.5%)
- Tumors ≥30 mm in one plane	25 (19%)	23 (37.5%)	2 (2.9%)
Preop Knosp, 0 both sides	43 (33%)	6 (10%)	37 (53.5%)
- 1-2	62 (48%)	36 (59%)	25 (37.5%)
- 3-4	25 (19%)	19 (31%)	6 (8.5%)
Preop tumor volume (n = 128)	2 missing	2 missing	
<1 ml	44 (34%)	-	44 (64%)
1-<10 ml	62 (48%)	40 (65%)	22 (32%)
≥10 ml	22 (17%)	19 (31%)	3 (4.3%)

### 3.4 Preoperative imaging and tumor characteristics

Preoperative MRI was available in 129 cases, and volumetric measurements in 128 cases (Table 2). Tumor diameter ranged from MRI-negative lesions to 52 mm in single largest plane. Overall, 100 tumors (77%) were macroadenomas (≥10 mm), including 25 tumors measuring ≥30 mm, and 22 tumors (17%) with preoperative volume ≥10 mL. Parasellar invasion (Knosp grades 3–4) was present in 25 cases (19%), occurring predominantly in tumors with volume ≥10 mL (14/22).

### 3.5 Postoperative imaging

Immediate postoperative MRI was performed in 128 patients. Radiological absence of residual tumor was reported in 66 cases (51%), while 18 cases (14%) demonstrated definite (n = 9) or possible (n = 9) residual tumor. The first routine follow-up MRI at approximately three months postoperatively was not performed in 27 functional cases due to remission status at endocrinological follow-up.

Overall, 73 of the 130 cases (56%) were radiologically assessed as having no residual tumor on 3-month follow-up MRI, whereas 22 patients (17%) had radiologically confirmed residual tumor (Table 3A). Residual tumor was more frequently observed in reoperations, invasive tumors (Knosp grades 3-4), and lesions with preoperative tumor volume >10 mL. Five non-functional cases had a delayed follow-up MRI (7–13 months) which was included in residual tumor assessment. Radiological and endocrine outcomes are reported separately to account for differences in follow-up strategy for functional adenomas (Table 3A, Table 3BA and 3B).

**Table 3A:** Radiological outcome at approximately three months postoperatively.

Outcome	All cases (n = 130)	NFPA (n = 61)	FPA (n = 69)	Previous surgery (n = 26)
Residual tumor	22 (17%)	12 (20%)	10 (14.5%)	7 (27%)
No residual tumor	73 (56%)	44 (72%)	29 (42%)	11 (42%)
Uncertain	4 (3%)	3 (5%)	1 (1.5%)	2 (7.5%)
MRI not performed^a^	27 (21%)	–	27 (39%)	5 (19%)
Missing	4 (3%)	2 (3%)	2 (3%)	1 (4%)

a MRI not performed due to biochemical remission in functional adenomas.

**Table 3B:** Early postoperative endocrine remission of FPAs.

Outcome	Functional tumors (n = 69)
Overall biochemical remission	45/69 (65%)
Acromegaly	27/42 (64%)
Cushing	14/17 (82%)
Prolactinoma	3/7 (42%)
Other functionality	1/3

Not in remission	21/69 (30%)

### 3.6 Endocrine outcomes

Early postoperative remission was achieved in 45/69 cases (65%; Table 3B) of which 39 were primary surgeries. The majority (n = 36) of remission cases had preoperative tumor volume <1 ml, 22 tumors were microadenomas, 2 were MRI-negative, and none (n = 0) demonstrated preoperative extensive cavernous sinus invasion (Knosp grades 3–4).

Among 17 patients with Cushing's disease, 14 (82%) achieved early biochemical remission within 7 days of surgery. However, most patients with Cushing's disease remained on glucocorticoid replacement therapy following endocrinological 3-month assessment, reflecting expected postoperative hypothalamic-pituitary-adrenal axis suppression rather than persistent hypercortisolism. Biochemical remission criteria were fulfilled in 27 of 42 patients with acromegaly (64%; Table 3B). One additional acromegaly-patient underwent delayed endocrine assessment 5.5 months postoperatively due to intercurrent illness, and fulfilled remission criteria at that time. Of the 7 patients undergoing surgery for prolactinoma, 3 (42%) had serum prolactin levels within normal range at the 3-month mark. One TSH-secreting adenoma was assessed as being in remission, whereas short-term remission status could not be determined in the 2 remaining cases.

Novel pituitary insufficiency requiring hormonal substitution at 3-month follow-up occurred in 19 patients (15%; Table 1). Within this group, 6 (5%) had a novel anterior deficiency including the corticotrope axis, and 9 (7%) had novel arginine-vasopressin deficiency (AVP-D). This includes 2 cases (1.5%) with novel complete anterior and posterior pituitary failure (Corticotrope and AVP-D). However, from the initial 9 AVP-D cases, only 6 (5%) still had AVP-D at 1-year follow-up. New pituitary dysfunction occurred predominantly in patients with non-functioning adenomas (n = 13). In one case, neither failure nor remission could be determined at the 3-month mark. One case was missing follow-up entirely (death).

### 3.7 Surgical complications

Postoperative CSF leak requiring surgical repair occurred in 7 patients (5.4%). Meningitis was diagnosed in 7 patients (5.4%; Table 1). Of these, 5 (4%) had CSF pleocytosis diagnosed by lumbar puncture and 3 had microbiological CSF investigation revealing fungal (n = 1) or bacterial (n = 2) etiology. The single case of fungal CNS-infection with Candida was seen several months after tumor resection, following several CSF-related interventions. Meningitis overlapped with CSF leak in 6 cases, resulting in 8 patients experiencing either or both complications (6.2%). All affected patients had moderate or high-grade intraoperative CSF leakage; 4/6 patients with combined CSF leak and meningitis had undergone previous pituitary surgery.

Epistaxis occurred in 6 patients (4.6%). One required surgical intervention beyond nasal packing. Other rare postoperative complications (each n = 1) included intratumoral hemorrhage requiring reoperation, ischemic infarction secondary to vasospasm with combined SIADH/DI, postoperative psychosis, infection in the abdominal fat-graft site, and systemic infection without an identified focus. One elderly, comorbid patient died approximately 3 months postoperatively after reoperation for postoperative hematoma and subsequent medical decline complicated by recurrent systemic infections. Death was considered multifactorial and not attributable to ICG administration.

## 4 Discussion

This prospective single-center study represents, to our knowledge, the largest clinical series to date evaluating intraoperative ICG fluorescence in endoscopic transsphenoidal surgery for pituitary adenomas and the first systematic assessment of continuous NIR/WL overlay visualization using a second-generation fluorescence endoscope. The principal finding is that continuous overlay ICG fluorescence can be implemented safely and reproducibly within an established endoscopic pituitary surgery workflow without disrupting intraoperative visualization or surgical orientation.

Previous studies of ICG in ETSS for PA have been limited to small cohorts and first-generation systems requiring alternating visualization modes, precluding continuous fluorescence-guided resection (Olesrud et al., 2025). In contrast, the present study demonstrates that continuous ICG fluorescence can be integrated into an established ETSS workflow across a broad spectrum of cases, including reoperations and invasive tumors.

A recent systematic review identified only 193 previously reported patients undergoing endoscopic ICG-guided pituitary surgery. Patients were identified across 15 studies, most utilizing first-generation systems requiring intermittent switching between white-light and fluorescence modes (Olesrud et al., 2025). The cohort presented in this paper therefore substantially expands the available clinical experience. To our knowledge, it represents the first systematic evaluation of continuous WL/NIR overlay fluorescence throughout routine pituitary adenoma resection. Unlike first-generation fluorescence systems, continuous overlay visualization permits simultaneous assessment of anatomical detail and fluorescence information without repeated mode switching, potentially allowing perfusion-based tissue characterization throughout resection.

The heterogeneous composition of the present cohort may also provide insight into clinical scenarios in which continuous fluorescence guidance could be most relevant. Potential applications may include identification of compressed or thinned residual pituitary gland tissue during resection of macroadenomas, differentiation between functional microadenomas and adjacent normal gland tissue, and distinction between tumor and residual gland in reoperations where scarring and distorted anatomy may limit conventional visual tissue discrimination.

However, we emphasize that these potential applications remain hypothesis-generating, and were not formally, nor objectively, evaluated in the current study.

While these observations suggest several potential avenues for future investigation, establishing clinical utility requires first demonstrating that the technology can be implemented safely and consistently in routine practice. Accordingly, the primary focus of the present study was feasibility rather than efficacy.

In the present study, feasibility extended beyond technical functionality and safety. Importantly, the technology could be incorporated into routine operative workflows, across a heterogeneous spectrum of surgical cases. Furthermore, implementation did not require surgeons to abandon fluorescence guidance, interrupt resection for repeated mode switching, or substantially modify their established endoscopic technique.

### 4.1 Interpretation of continuous overlay ICG fluorescence

Continuous ICG fluorescence adds a new dimension to intraoperative visualization that differs fundamentally from intermittent fluorescence assessment. Rather than serving as a confirmatory tool, fluorescence becomes a dynamic background signal reflecting tissue perfusion throughout resection. Importantly, surgeons reported that switching back to standard white-light illumination was not necessary for any of the cases. This indicates that NIR/WL overlay did not compromise intraoperative orientation or anatomic detail.

Although the technique requires surgeons to interpret fluorescence intensity and temporal dynamics of the ICG signal, no significant technical barriers or workflow interruptions were encountered. While the present study was not designed to quantify a learning curve, the absence of reported conversion to white-light-only visualization suggests that adaptation to continuous overlay is feasible in experienced endoscopic teams.

### 4.2 Variability of fluorescence signal and ICG formulation

Intraoperative fluorescence intensity varied between patients and was influenced by dose, timing, tissue perfusion, and viewing geometry, consistent with prior reports of endoscopic ICG use in pituitary surgery (Olesrud et al., 2025). In most cases, the normal pituitary gland demonstrated stronger fluorescence than adenoma tissue, in line with previous hypotheses based on differences in vascularity (Litvack et al., 2012). However, tumor fluorescence was also observed in a subset of cases, possibly reflecting heterogeneity in tumor microvasculature and subtype-specific perfusion patterns, as previously described (Di Ieva et al., 2013).

These findings indicate that intraoperative fluorescence cannot be uniformly interpreted as a surrogate marker of normal gland tissue. Interpretation of fluorescence signal therefore requires contextual anatomical judgment, particularly during early clinical adoption of the technique.

During the study period, the ICG formulation was changed due to supply constraints. Although no formal comparative analysis was conducted, variations in perceived fluorescence intensity and signal persistence were observed across cases. Experimental data indicate that solvent composition and concentration can influence the absorption and emission characteristics of ICG in-vitro (Haritoglou et al., 2003), suggesting that commercially available formulations may not be optically identical despite uniform clinical labeling. Systematic clinical evaluation of formulation-dependent fluorescence characteristics represents an important and currently underexplored area for further investigation.

### 4.3 Did ICG influence outcome?

The observed complication profile was comparable to historical endoscopic series from our center, suggesting that integration of continuous ICG visualization did not introduce additional procedural risk (Halvorsen et al., 2014). Rates of postoperative CSF leak requiring repair were within expected ranges for endonasal endoscopic skull-base surgery (Consortium, 2021; Lobatto et al., 2018). Using different definitions, meningitis has been reported at rates spanning from 0 to almost 10% across studies (Lobatto et al., 2018). The observed meningitis rate in the current group was slightly higher than that reported in a prior institutional series (Halvorsen et al., 2014). This difference is likely influenced by broad case definitions and extended surveillance in the present study, including clinically suspected cases treated empirically and a single delayed case of Candida meningitis. When applying more restrictive criteria commonly used in the literature - meningitis occurring within 30 days postoperatively with biochemical verification by CSF-analysis - the incidence in the current cohort was 2.3% (3 of 130 cases).

Short-term endocrine outcomes, particularly remission rates in acromegaly, were favorable relative to historical data but cannot be attributed to fluorescence guidance alone. These outcomes likely reflect cumulative effects of surgical technique evolution, experience, and perioperative management rather than ICG use per se. There is no clear consensus on a single clinical definition of postoperative remission, and endocrinological remission rates will vary according to applied cut-off values and definitions for remission (Falch et al., 2023; Ramm-Pettersen et al., 2015). Furthermore, assessment of novel pituitary failure at 3 months after surgery is too premature to reflect long-term outcomes. Accordingly, the present data should not be interpreted as evidence for improved extent of resection or long-term endocrine remission.

### 4.4 Impact of case mix and surgical indication

Interpretation of pituitary surgery outcomes requires careful consideration of institutional case mix and indication thresholds. The present cohort intentionally included all consecutive consenting patients, encompassing primary and repeat surgeries, functional and non-functional adenomas, and a substantial proportion of invasive Knosp grade 3-4 tumors. This inclusive approach reflects real-world clinical practice, but limits comparability with series restricted to primary or non-invasive lesions. Reoperations, tumors of larger volumes and cavernous sinus invasion are well known to carry lower remission rates and higher complication risk (Almeida et al., 2018; Castle-Kirszbaum et al., 2023; Lobatto et al., 2018). These factors must be considered when contextualizing the results.

### 4.5 Limitations

This study is limited by its observational, single-center design and absence of a parallel control group.

Imaging and endocrine follow-up results were limited to short-term follow-up at approximately three months postoperatively. Follow-up was heterogeneous in some areas, with variable MRI-contrast use and some imaging was performed externally. Completion of missing registry data by retrospective chart review introduces potential observer bias, and radiological assessments were subject to inter-reader variability. Fluorescence signal interpretation was not standardized or quantitatively assessed in this study. The present study was designed to assess the implementation, safety and feasibility of a surgical method, not to determine whether ICG fluorescence improves extent of resection, endocrine remission, or complication rates, and any such inference would be inappropriate.

Furthermore, ICG fluorescence was not universally implemented throughout the study period, introducing the possibility of selection bias. Although a review of non-ICG cases did not suggest systematic selection with regard to tumor subtype or complexity, such bias cannot be fully excluded in an observational implementation study.

Finally, the present study was not designed to evaluate the diagnostic accuracy of fluorescence for gland or tumor identification, nor to determine whether fluorescence guidance improves extent of resection, endocrine remission, or preservation of pituitary function.

### 4.6 Clinical implications and future directions

Continuous overlay fluorescence represents a safe and operationally practical visualization platform for endoscopic pituitary surgery, enabling real-time perfusion contrast without workflow interruption. The present study establishes the foundation for subsequent investigations including outcome effects, which should focus on matched comparative outcome analyses between ICG- and non-ICG-treated cohorts from the same institution, as well as histopathological correlation of intraoperative fluorescence with tumor and normal pituitary tissue.

Further work is needed to standardize ICG dosing strategies, characterize signal duration and re-dosing thresholds during prolonged resections, and evaluate formulation-dependent optical variability. Dedicated comparative studies are required to determine whether fluorescence guidance improves surgical decision-making or clinical outcomes in different settings, such as re-operations and variable tumor complexity*.* Such studies will be essential to determine whether fluorescence guidance during ETSS can meaningfully influence intraoperative decision-making and long-term surgical and endocrine outcomes. Regulatory restrictions under the European Medical Device Regulation currently limit access to second-generation fluorescence systems, underscoring the importance of real-world implementation data such as those presented here.

## 5 Conclusions

This study provides the first large clinical evaluation of continuous NIR/WL overlay endoscopy in pituitary adenoma surgery. No dye- or device-related complications were observed, and overall morbidity mirrored established benchmarks. Continuous overlay ICG fluorescence visualization can be safely integrated into routine endoscopic practice and establishes a robust platform for future comparative and translational studies.

## Ethics declaration

This study was conducted in accordance with the 1964 Helsinki declaration and its later amendments and according to the ethical standards of Oslo University Hospital.

## Human ethics and consent to participate declarations

All included subjects gave written consent for participation and publication of their data.

## IRB approval

Written informed consent for intraoperative ICG administration and inclusion of clinical data for research and publication was obtained from all patients. The study protocol was approved by the Regional Committee for Medical and Health Research Ethics (REK no. 399198) and the institutional Privacy Protection Officer at Oslo University Hospital (PVO no. 22/21929).

Exemption from the conformity assessment procedure with regards to the endoscopic equipment was granted by the Norwegian Medicines Agency (NoMA, ref 22/09780-2).

## Declaration of generative AI and AI-assisted technologies in the manuscript preparation process

During the preparation of this work, some of the authors used the AI-tool ChatGPT version 5.2 (OpenAI) and Google AI-assistant (Gemini 2.5 Flash/3) in order to improve the language of the paper. After using this tool/service, the authors reviewed and edited the content as needed and take full responsibility for the content of the published article.

## Funding

This study was supported by the South-Eastern Norway Regional Health Authority (grant number 2024021).

## Declaration of competing interest

The authors declare the following financial interests/personal relationships which may be considered as potential competing interests:Ida Olesrud reports financial support was provided by South-Eastern Norway Regional Health Authority. If there are other authors, they declare that they have no known competing financial interests or personal relationships that could have appeared to influence the work reported in this paper.
